# Role and benefits of infectious diseases specialists in the COVID-19 pandemic: Multilevel analysis of care provision in German hospitals using data from the Lean European Open Survey on SARS-CoV-2 infected patients (LEOSS) cohort

**DOI:** 10.1007/s15010-024-02362-2

**Published:** 2024-08-16

**Authors:** Lene T. Tscharntke, Norma Jung, Frank Hanses, Carolin E. M. Koll, Lisa Pilgram, Siegbert Rieg, Stefan Borgmann, Susana M. Nunes de Miranda, Margarete Scherer, Christoph D. Spinner, Maria Rüthrich, Maria J. G. T. Vehreschild, Michael von Bergwelt-Baildon, Kai Wille, Uta Merle, Martin Hower, Katja Rothfuss, Silvio Nadalin, Hartwig Klinker, Julia Fürst, Ingo Greiffendorf, Claudia Raichle, Anette Friedrichs, Dominic Rauschning, Katja de With, Lukas Eberwein, Christian Riedel, Milena Milovanovic, Maximilian Worm, Beate Schultheis, Jörg Schubert, Marc Bota, Gernot Beutel, Thomas Glück, Michael Schmid, Tobias Wintermantel, Helga Peetz, Stephan Steiner, Elena Ribel, Harald Schäfer, Jörg Janne Vehreschild, Melanie Stecher, the LEOSS Study Group

**Affiliations:** 1 Klinik I für Innere Medizin, Centrum für Integrierte Onkologie Aachen Bonn Köln Düsseldorf, Universität zu Köln, Medizinische Fakultät und Uniklinik Köln, Köln, Germany; 2 Universitätsklinikum Regensburg, Zentrale Notaufnahme, Regensburg, Germany; 3 Klinik I Für Innere Medizin, Universität Zu Köln, Medizinische Fakultät Und Uniklinik Köln, Köln, Germany; 4 Deutsches Zentrum Für Infektionsforschung (DZIF), Partnerstandort Bonn-Köln, Cologne, Germany; 5 Klinik Für Innere Medizin, Hämatologie Und Onkologie, Universitätsklinikum Frankfurt, Goethe Universität Frankfurt, Frankfurt am Main, Germany; 6 Department of Nephrology and Medical Intensive Care, Charité - Universitätsmedizin Berlin, Berlin, Germany; 7 Abteilung Infektiologie, Klinik Für Innere Medizin II, Universitätsklinikum Freiburg, Medizinische Fakultät, Albert-Ludwigs-Universität Freiburg, Freiburg, Germany; 8 Abteilung Klinische Infektiologie Und Hygiene, Klinikum Ingolstadt, Ingolstadt, Germany; 9 Technische Universität München, Fakultät Für Medizin, Klinikum Rechts Der Isar, Klinik Und Poliklinik Für Innere Medizin II, Munich, Germany; 10 Klinik für Innere Medizin, Infektiologie, Universitätsklinikum Frankfurt, Goethe Universität Frankfurt, Frankfurt am Main, Germany; 11 LMU Klinikum München, Medizinische Klinik und Poliklinik III, Munich, Germany; 12 Universität Bochum, Universitätsklinik für Hämatologie, Onkologie, Hämostaseologie und Palliativmedizin, Minden, Germany; 13 Abteilung für Gastroenterologie und Infektiologie, Universitätsklinikum Heidelberg, Heidelberg, Germany; 14 Klinikum Dortmund gGmbH, Klinik für Pneumologie, Infektiologie und internistische Intensivmedizin, Klinikum der Universität Witten/Herdecke, Dortmund, Germany; 15 Abteilung für Gastroenterologie, Hepatologie und Endokrinologie, Robert-Bosch-Krankenhaus Stuttgart, Stuttgart, Germany; 16 Universitätsklinik für Allgemein-, Viszeral- und Transplantationschirurgie, Universitätsklinikum Tübingen, Tübingen, Germany; 17 Universitätsklinikum Würzburg, Medizinische Klinik und Poliklinik II, Infektiologie, Würzburg, Germany; 18 Medizinische Klinik I, Universitätsklinikum Erlangen, Erlangen, Germany; 19 Kliniken Maria Hilf Mönchengladbach GmbH, Innere Medizin I, Klinik für Hämatologie, Onkologie und Gastroenterologie, Mönchengladbach, Germany; 20 Tropenklinik Paul-Lechler Krankenhaus Tübingen, Tübingen, Germany; 21 Universitätsklinikum Schleswig–Holstein, Campus Kiel, Klinik für Innere Medizin I, Kiel, Germany; 22 Klinik IB für Innere Medizin, Bundeswehrkrankenhaus Koblenz, Koblenz, Germany; 23 Universitätsklinikum Carl Gustav Carus Dresden an der TU Dresden, Klinische Infektiologie, Dresden, Germany; 24 Klinikum Leverkusen, Medizinische Klinik IV, Leverkusen, Germany; 25 Helios Klinikum Pirna, Pirna, Germany; 26 Malteser Krankenhaus St. Franziskus Hospital Flensburg, Flensburg, Germany; 27 Oberlausitz-Kliniken gGmbH/Krankenhäuser Bautzen und Bischofswerda, Bischofswerda, Germany; 28 Marien Hospital Herne, Universitätsklinikum der Ruhr-Universität Bochum, Bochum, Germany; 29 Elblandklinikum Riesa, Riesa, Germany; 30 Agaplesion Bethesda Krankenhaus Bergedorf, Hamburg, Germany; 31 Medizinische Hochschule Hannover Klinik für Hämatologie, Hämostaseologie, Onkologie und Stammzelltransplantation, Hannover, Germany; 32 Kliniken Südostbayern AG Trostberg, Trostberg, Germany; 33 Medizinische Klinik I Hegau-Bodensee-Klinikum Singen, Singen, Germany; 34 Krankenhaus der Augustinerinnen Köln, Köln, Germany; 35 Die Klinik in Preetz, Preetz, Germany; 36 St. Vincenz KH Limburg, Limburg, Germany; 37 Hunsrück Klinik Kreuznacher Diakonie, Simmern, Germany; 38 SHG Kliniken Völklingen, Med. Klinik II, Pneumologie, Thorakale Onkologie, Infektiologie, Völklingen, Germany; 39 Klinik für interdisziplinäre Intensivmedizin, Vivantes Humboldt-Klinikum Berlin, Berlin, Germany

**Keywords:** Infectious diseases medicine, COVID-19, Pandemic, Healthcare research, Healthcare quality, Quality indicators

## Abstract

**Purpose:**

This study investigates the care provision and the role of infectious disease (ID) specialists during the coronavirus disease-2019 (COVID-19) pandemic.

**Methods:**

A survey was conducted at German study sites participating in the Lean European Open Survey on SARS-CoV-2 infected patients (LEOSS). Hospitals certified by the German Society of Infectious diseases (DGI) were identified as ID centers. We compared care provision and the involvement of ID specialists between ID and non-ID hospitals. Then we applied a multivariable regression model to analyse how clinical ID care influenced the mortality of COVID-19 patients in the LEOSS cohort.

**Results:**

Of the 40 participating hospitals in the study, 35% (14/40) were identified as ID centers. Among those, clinical ID care structures were more commonly established, and ID specialists were always involved in pandemic management and the care of COVID-19 patients. Overall, 68% (27/40) of the hospitals involved ID specialists in the crisis management team, 78% (31/40) in normal inpatient care, and 80% (28/35) in intensive care. Multivariable analysis revealed that COVID-19 patients in ID centers had a lower mortality risk compared to those in non-ID centers (odds ratio: 0.61 (95% CI 0.40–0.93), p = 0.021).

**Conclusion:**

ID specialists played a crucial role in pandemic management and inpatient care.

**Supplementary Information:**

The online version contains supplementary material available at 10.1007/s15010-024-02362-2.

## Introduction

The coronavirus disease-2019 (COVID-19) pandemic posed major challenges to societies, healthcare systems, and economies worldwide [1]. At the onset of the pandemic, health policy and care-related decisions had to be made initially without established evidence due to the lack of longitudinal data for evidence based and purposive pandemic management [1, 2]. Since the beginning of the pandemic in Germany, the Robert Koch Institute (RKI) has regularly reported infection rates, demographic characteristics, as well as the guidelines on the diagnosis, hygiene, therapy and infection control of COVID-19 [3]. These therapy guidelines and recommendations were based, among other sources, on guidance from the Standing Working Group of Competence and Treatment Centres for high consequence infectious diseases (STAKOB), as well as statements from the German Society of Infectious Diseases (DGI) and the German Society of Paediatric Infectious Diseases (DGPI) [3, 4]. Since February 2021, the S3 guideline “Recommendations for the inpatient treatment of patients with COVID-19”, has also provided specific recommendations, with the DGI playing a leading role in its establishment [5]. In addition to specialist expertise, both clinical trials and cohort studies are of great importance in the field of public health and healthcare research [6]. At the beginning of the COVID-19 pandemic, the Lean European Open Survey on SARS-CoV-2 infected patients (LEOSS) was initiated with the support of the German Centre for Infection Research (DZIF), the DGI and the European Society of Clinical Microbiology and Infectious Diseases (ESCMID) [7, 8]. LEOSS is a cohort providing a comprehensive database on the acute clinical course of COVID-19 patients. The data are made available to the scientific community following an open science approach, with the aim of rapidly generating an evidence base for decision-makers [8].

However, in addition to clinical data on the course of the disease, structural-level data are needed to enable conclusions for a higher quality and more efficient medical care [2]. The objective of this study is to evaluate care provision and the role of clinical ID medicine in Germany, in order to provide important evidence for health policy and care-related decisions on current and future challenges in ID medicine.

## Methods

### Data collection and study design

We considered two methodological levels in our study: (I) the structural characteristics of the hospitals examined and (II) the socio-demographic and clinical course data of patients from the LEOSS cohort.

#### I. Data collection at structural-level

Hospitals in Germany that were study sites of the LEOSS cohort at the beginning of the pandemic were examined. In order to identify the structural characteristics of these hospitals, a questionnaire was send to one head physician or one senior physician from a total of the 70 German sites participating in LEOSS in August 2020.

For the selection of the interviewed physicians, it was crucial that they held a leading role within the COVID-19 pandemic at that time and were project coordinators of the LEOSS cohort at their respective study side. The data collection was conducted retrospectively for the period from March 1st to April 30th, 2020, using an electronic questionnaire: Items were collected to record (1) the clinical ID care provision, (2) the structure and organization of healthcare during the pandemic, and (3) the integration of clinical ID medicine in pandemic management and care (Supplement 1). Among these items, essential elements within the clinical-infectiological care provision represented by ten structure indicators, as well as memberships in ID medical associations and certifications were considered [9, 10]. The questionnaire was made available through the established cohort platform ClinicalSurvey.net, which was developed at the University Hospital of Cologne and is operated by the company Questback in Oslo, Norway.

#### II. Data collection at patient-level

The patient-level analyses were based on data from the anonymous and retrospective multicenter LEOSS cohort, which was established in March 2020 [7]. LEOSS collects sociodemographic and clinical follow-up data of patients with polymerase chain reaction (PCR)-confirmed SARS-CoV-2 infection.

### Statistical analysis

#### I. Analyses at structural-level

The data at the structural-level were presented descriptively. Median (M) and interquartile range (IQR) were calculated. Hospitals with externally validated ID expertise were identified and defined as ID centers. The identification was based on certification as a DGI-center, which is considered a structure indicator of clinical ID care provision [9]. A comparison was then made between ID and non-ID centers: the items collected from the areas (1), (2) and (3) described in the method section above, as well as the structure indicators of clinical ID care provision, were compared using the Chi-square test or Fisher's exact test. The results were presented in absolute and relative numbers. The level of significance was p < 0.05.

#### II. Analyses at patient-level

In a second step, the questionnaire data (structure-level) were merged with those of the LEOSS cohort (patient-level). Univariate and multivariable binary logistic regression models were performed. The primary endpoint of the analyses was mortality during the acute course of SARS-CoV-2 infection (deceased vs. not deceased) of patients in the LEOSS cohort who were ≥ 18 years and were hospitalized at the study sites between March 1, 2020, and April 30, 2020. The multivariable model was adjusted for potential confounders, and its robustness was tested in subgroup analyses. Results were presented as odds ratios (OR) with 95% confidence interval (95% CI). Detailed information on the selection of confounders and the final regression model is provided in Supplement 2. All statistical analyses were performed using IBM^®^ SPSS^®^ statistical software, version 28.0 (Released 2021, Armonk, NY: IBM Corp).

## Results

### Description of the hospital characteristics and the clinical ID care provision

**Fig. 1:**
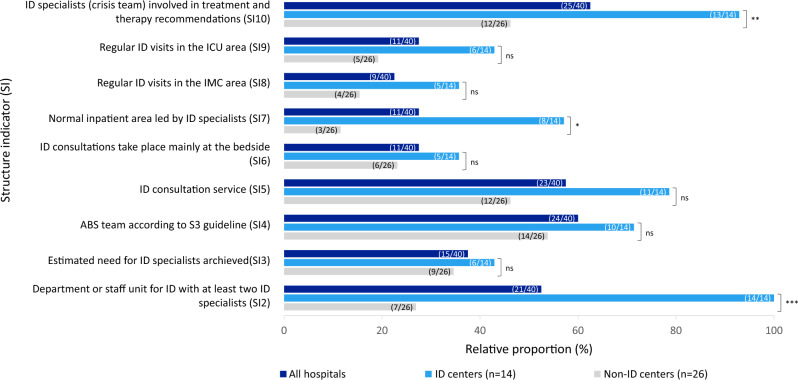
Established structure indicators of clinical ID care provision at all hospitals (n = 40) and comparison between ID (n = 14) and non-ID centers (n = 26). Explanation: Comparison using Chi-square test or Fisher's exact test. *p-value* p-value in the Chi-square test or Fisher's exact test (significance level *p ≤ 0.05; **p ≤ 0.005; ***p < 0.001, ns = not significant), *ABS* Antibiotic Stewardship, *ID* infectious diseases, *IMC area* Intermediate Care area, *ICU area* Intensive Care Unit area; *SI2*: The hospital has a department or staff unit for ID in which at least two ID physicians work who are qualified as ID specialists according to the German Society for Infectiology (DGI) or state medical association. *SI3:* Estimated need for ID specialists per number of beds according to Kern et al. is achieved; *SI4*: ABS team according to S3 guideline; *SI5:* There is an ID consultation service; *SI6:* ID consultations take place mainly at the bedside; *SI7*: Normal inpatient areas are led by ID specialists; *SI8:* Regular ID visits in the IMC area; *SI9:* Regular ID visits in the ICU area; *SI10:* ID specialists are involved in treatment and therapy recommendations within the framework of the crisis team

In the survey with the physicians from each LEOSS study site, 57% (40/70) participated, providing a diverse set of hospital-level information. The hospitals varied in both their bed capacity (range 90–2100, M 845, IQR 974) and the number of departments (range 1–65, M 20.5, IQR 28). Of the participating LEOSS sites, 40% (16/40) were university hospitals, 35% (14/40) were ID centers, 20% (8/40) were DZIF membership institutions, and 10% (4/40) were STAKOB centers (multiple selection possible). Among all hospitals, 30% (12/40) were both ID and university centers. Considering only the university centers, 75% (12/16) were ID centers and 25% (4/16) were non-ID centers. We identified 14 ID centers and 26 non-ID centers, highlighting differences in bed capacity: 740–2100 beds (M 1400, IQR 421) in ID-centers, compared to 90–1991 beds (M 520.5, IQR 710) in non-ID centers. Structure indicators of the clinical ID care provision were established in 88% (35/40) of the hospitals (Fig. 1). The provision of care varied significantly between ID centers and non-ID centers, particularly in terms of the presence of an ID department or staff unit (100%, 14/14 vs. 27%, 7/26, p < 0.001) and the authorization for further training in ID according to the state medical association (LÄK) (100%, 14/14 vs. 35%, 9/26, p < 0.001). Additional information regarding the clinical ID care provision is available in Table 1.

**Table 1:** Clinical ID care provision surveyed at the LEOSS study sites in addition to the structural indicators in Fig. 1

	All hospitals	ID centers	Non-ID centers	p-value
*Total*	40	35% (14/40)	65% (26/40)	–
*Externally validated ID expertise*				
DZIF membership institution	20% (8/40)	50% (7/14)	4% (1/26)	**0.001**
STAKOB-center	10% (4/40)	21% (3/14)	4% (1/26)	0.115
*Staff/Team/Infrastructure*				
ID beds on own ward	45% (18/40)	71% (10/14)	31% (8/26)	**0.021**
ID beds as occupancy beds	13% (5/40)	29% (4/14)	4% (1/26)	**0.043**
Neither a department or staff unit, an ABS team nor a consultation service has been established in the hospital	8% (3/40)	0% (0/14)	12% (3/26)	0.539
ABS team in alternative form of organisation	20% (8/40)	21% (3/14)	19% (5/26)	1.000
Explicit isolation rooms (room class II, negative pressure, airlock) according to DGKH guidelines. (100% of the isolation rooms were used for COVID-19 patients)	60% (24/40)	79% (11/14)	50% (13/26)	0.101
Diagnostic laboratory with availability of all modern detection methods for the diagnosis of infectious agents and infections in the hospital	78% (31/40)	100% (14/14)	65% (17/26)	**0.016**
*Further education and training*				
Further training authorisation in ID according to LÄK is available	58% (23/40)	100% (14/14)	35% (9/26)	** < 0.001**
Regular ID training (weekly or monthly)	35% (14/40)	79% (11/14)	12% (3/26)	** < 0.001**
*Involvement of ID specialists in the inpatient care of COVID-19 patients*				
Involvement in the normal inpatient setting	78% (31/40)	100% (14/14)	65% (17/26)	**0.016**
Involvement in the IMC area, if available	78% (18/23)	100% (9/9)	64% (9/14)	0.116
Involvement in the ICU area, if available	80% (28/35)	100% (13/13)	68% (15/22)	**0.031**

Explanation: Group comparison between ID and non-ID centers by means of Chi-square or Fisher's Exact test. *p-value* p-value in Chi-square or Fisher's Exact test (significance level p < 0·05), *ABS* Antibiotic Stewardship, *DGKH* German Society for Hospital Hygiene, *DZIF* German Centre for Infection Research, *ID* Infectious diseases, *IMC area* Intermediate Care area, *ICU area* Intensive Care area, *LÄK* State Medical Association, *STAKOB* Standing Working Group of Competence and Treatment Centers for high consequence infectious diseases

### Structure and organization of care during the pandemic

During the pandemic, patient care in the inpatient setting was carried out in 88% (35/40) according to the recommendations of the RKI: To prevent the spread of the SARS-CoV-2 virus, strict separation was enforced between SARS-CoV-2 positive patients, suspected cases, and SARS-CoV-2 negative patients. There was no significant difference regarding separation strategies between ID centers and non-ID centers (86%, 12/14 vs. 89%, 23/26, p = 1.00). In all normal inpatient areas of the hospitals, a separate ward or area for the care of COVID-19 patients and suspected cases was established. However, this measure was only implemented in 88% (35/40) of the intensive care unit (ICU), in 58% (23/40) in the intermediate care unit (IMC), in 35% (14/40) in the pediatric area and in 28% (11/40) in the palliative care areas. Specific isolation rooms (room class II, negative pressure, airlock) according to the guidelines of the German Society for Hospital Hygiene (DGKH) existed in 60% (24/40) of the hospitals and were exclusively used for COVID-19 patients [11]. New care structures were also established in the outpatient and pre-hospital settings during the COVID-19 pandemic: 45% (18/40) of the participating centers had separate COVID-19 ambulances, and 80% (32/40) had dedicated test centers (e.g. tents or containers). A contribution to research on the emerging ID was made in all hospitals through participation in study registries, in 40% (16/40) through the inclusion of patients in interventional clinical studies and in 50% (20/40) through the collection of biomaterial. ID centers were significantly more likely to be involved in research projects compared to non-ID centers (clinical trials: 79%, 11/40 vs. 19%, 5/26, p < 0.001; biomaterial: 86%, 12/14 vs. 31, 8/26, p = 0.002).

**Fig. 2:**
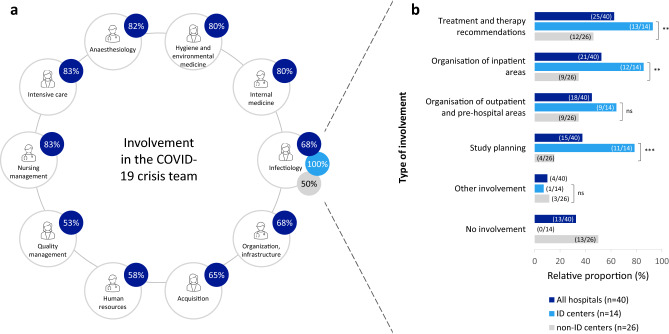
**a** (left): Departments involved in the COVID-19 crisis team in all hospitals (n = 40). Explanation: Only departments that were involved in at least 50% (20/40) are shown. Other departments involved were Microbiology (45%, 18/40), Surgery (35%, 14/40), Paediatrics (33%, 13/40), Virology (30%, 12/40), Palliative medicine (23%, 9/40), Neurology (18%, 7/40), Transfusion medicine (15%, 6/40), Infectious disease epidemiology (8%, 3/40), Finance department (33%, 13/40), Cost department (25%, 10/40), Other (35%, 14/40). **b** (right): Type of involvement of ID specialists in the COVID-19 crisis team. Explanation: Shown is the type of involvement in all hospitals (n = 40) as well as the comparison between ID (n = 14) and non-ID centers (n = 26) using the Chi-square test or Fisher's exact test, multiple selection possible. *p-value* p-value in the Chi-square test or Fisher's exact test (significance level *p ≤ 0.05; **p ≤ 0.005; ***p < 0.001, ns = not significant), *ID* infectious diseases

The healthcare management was interdisciplinary and collaborative. In the inpatient setting, 19 different specialized disciplines were involved in direct patient care. The crisis teams were also organized on in interdisciplinary manner. Besides ID medicine, the departments of intensive care medicine (83%, 33/40), internal medicine (80%, 32/40), hygiene and environmental medicine (80%, 32/40), as well as nursing management (83%, 33/40) and several other departments were involved (Fig. 2a). Communication and cooperation also took place across clinics: An interdisciplinary exchange of experts took place in over half of all hospitals (55%, 22/40) to discuss treatment options for SARS-CoV-2 patients with a severe course of disease. The proportion in ID centers was 43% (6/14), which was lower than in non-ID centers (62%, 16/26, p = 0.257). Expertise was obtained from a university hospital in 38% (15/40) of cases and from an ID or STAKOB center in 23% (9/40). Discharge of patients with a severe course of disease occurred in 28% (11/40) of cases, with patients from non-ID centers being discharged more frequently (7%, 1/14 vs. 39%, 10/26, p = 0.061).

### Involvement of clinical ID medicine in pandemic management and care

**Fig. 3:**
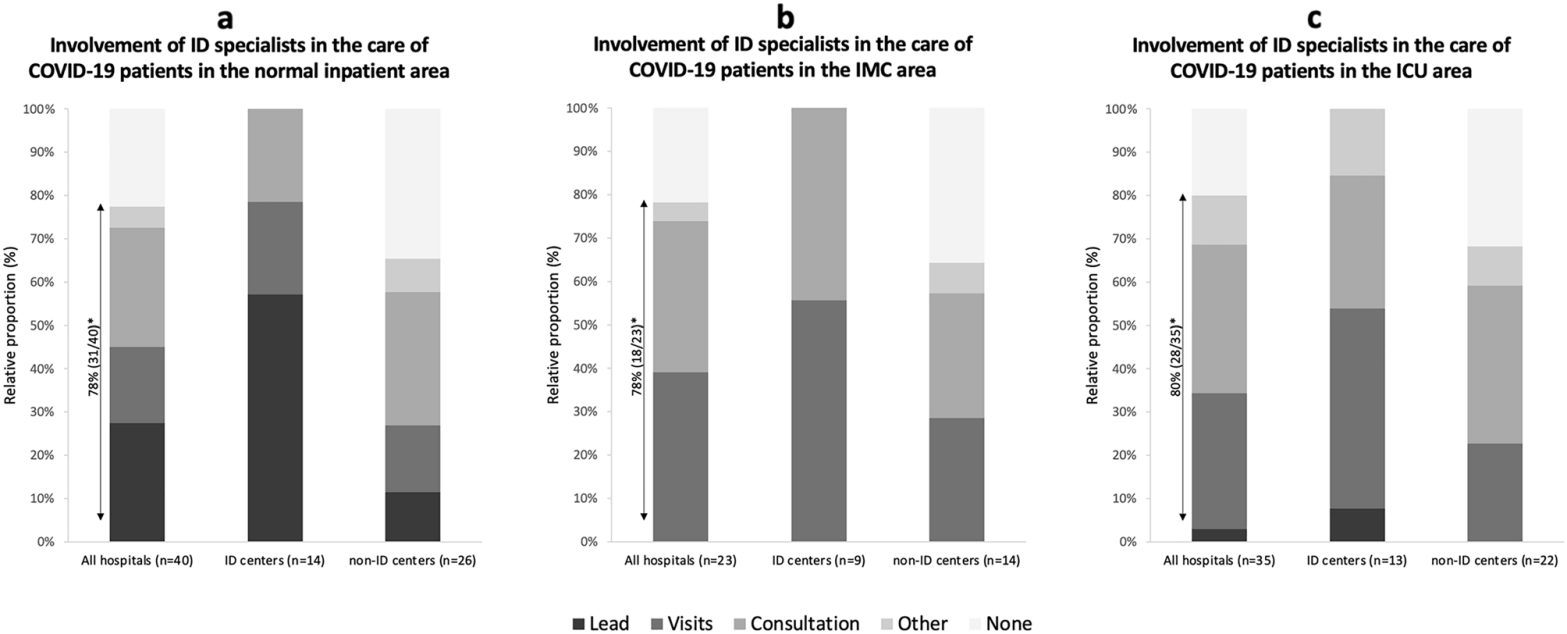
Type of involvement of ID specialists in the care of COVID-19 patients in **a** normal in patient area, **b** IMC area, and **c** ICU area. Explanation: Shown for all hospitals as well as ID and non-ID centers (if such areas existed in March/April 2020). Single choice, possible answers: A realed by ID specialists, regular ID visits, ID consultations servicewhen required, other, or no involvement. *ID* Infectiousdiseases, *IMC area* Inter mediate care area, *ICU area* Intensive care area. *Percentage of hospitals in which ID specialists were involved

The involvement of ID medicine in the crisis team and associated decision-making processes was found in 68% (27/40) of the hospitals. This concept was more frequently implemented in the 14 ID centers compared to non-ID centers (100%, 14/14 vs. 50%, 13/26, p = 0.001). ID specialists assumed various roles within the crisis management team. They were involved in organizing outpatient, pre-admission, and inpatient COVID-19 areas, providing treatment and therapy recommendations, as well as planning of research studies (see Fig. 2b). ID specialists played a key role in the care of COVID-19 patients in the inpatient setting. In the normal inpatient area, they were involved in the care in a total of 78% (31/40) of the hospitals. In ID centers, the normal inpatient area was significantly more often led by ID specialists (57%, 8/14 vs. 12%, 3/26, p = 0.007) compared to non-ID centers. In hospitals with an IMC area for COVID-19 patients, the rate of involvement was 78% (18/23), while in hospitals with an ICU area for COVID-19 patients, it was 80% (28/35) (Table 1). The type of involvement is shown in detail in Fig. 3a–c.

### Impact of clinical ID expertise on the treatment of patients in the LEOSS cohort

Overall, 3160 patients were enrolled in LEOSS over the study period, of which 63·4% (2004/3160) were treated at the 40 hospitals participating in this study. Among the 2004 included patients, 18·0% (361/2004) died during the acute course. A higher risk of death was found for older patients (> 85 years) compared to younger ones (≤ 45 years) (OR 21.30 (95% CI 8·14–55.76), p < 0.001), those with a critical compared to an uncomplicated stage of disease at diagnosis (OR 8.66 (95% CI 5·58–13.43), p < 0.001) as well as for men (OR 1.58 (95% CI 1.16–2.14), p = 0.003), and those with conditions such as kidney disease (OR 1.80 (95% CI 1.29–2.51), p < 0.001) and/or chronic pulmonary disease (OR 1.47 (95% CI 1.01–2.14), p = 0.042) (Table 2).

**Table 2:** Univariate and multivariable logistic regression analysis with the clinical endpoint mortality

	All patients	Univariate analysis		Multivariable analysis	
		OR (95%CI)	p-value	OR (95%CI)	p-value
*Total*	2004	–	–	–	–
*Age*					
< = 45	16.1% (323/2004)	Ref.		Ref.	
46–65	36.0% (722/2004)	3.50 (1.65–7.42)	0.001	2.33 (0.96–5.67)	0.063
66–85	39.9% (800/2004)	14.94 (7.28–30.64)	< 0.001	7.65 (3.15–18.58)	** < 0.001**
> 85	7.9% (159/2004)	34.28 (15.91–73.87)	< 0.001	21.30 (8.14–55.76)	** < 0.001**
*Gender*					
Female	41.7% (835/2004)	Ref.		Ref.	
Male	58.3% (1169/2004)	1.55 (1.22–1.97)	< 0.001	1.58 (1.16–2.14)	**0.003**
*Comorbidities*					
Cardiovascular diseases	56.1% (1096/1952)	4.09 (3.07–5.44)	< 0.001	1.26 (0.86–1.85)	0.236
Diabetes mellitus	19·0% (373/1959)	2.25 (1.73–2.93)	< 0.001	0.98 (0.70–1.38)	0.901
Chronic pulmonary disease	14·8% (290/1955)	1.87 (1.39–2.50)	< 0.001	1.47 (1.01–2.14)	**0.042**
Haematological or oncological disease	14.9% (291/1956)	1.79.(1·34–2·41)	< 0·001	1.38.(0·96–2·00)	0·083
Renal disease	16.6% (326/1960)	3.24.(2·48–4·23)	< 0·001	1.80.(1·29–2·51)	** < 0.001**
Neurological disease	22.5% (391/1735)	2.51.(1·93–3·25)	< 0·001	1.21.(0·87–1·69)	0.261
*Stage of disease at diagnosis*					
Uncomplicated	64.6% (1266/1959)	Ref.		Ref.	
Complicated	27.1% (530/1959)	3.70.(2·83–4·84)	< 0.001	2.67 (1.94–3.65)	** < 0.001**
Critical	8.3% (163/1959)	8.17 (5.68–11.73)	< 0.001	8.66 (5.58–13.43)	** < 0.001**
*Body-Mass-Index*					
< 18.5	2.6% (28/1085)	1.13 (0.44–2.88)	0.799	*	*
18·5–24.9	37.0% (401/1085)	Ref.		*	*
25–29.9	34.6% (375/1085)	1.04 (0.73–1·48)	0.848	*	*
30–34.9	16.3% (177/1085)	1.02 (0.65–1·59)	0.928	*	*
> 34.9	9.6% (104/1085)	1.24 (0.74–2·09)	0.412	*	*
*Characteristics of the hospitals*					
University center	63.9% (1280/2004)	0.81 (0.64–1.02)	0.078	1.63 (1.05–2.54)	**0.029**
ID center	60.0% (1203/2004)	0.66 (0.53–0.83)	< 0.001	0.61 (0.40–0.93)	**0.021**

Explanation: *CI*95% 95% confidence interval, *ID* infectious diseases, *OR* odds ratio, *Ref* reference category, *p-value* p-value for univariate or multivariable analysis (in the multivariable logistic regression analysis, confounders with a univariate significance level ≤ 0.2 were considered). Confounders considered at patient-level: age (in years), gender, comorbidities (no reference group, comorbidities were dichotomised, see appendix 2), stage of disease at diagnosis (see appendix 2), body mass index (in kg/m^2^, *no inclusion because univariate sig. > 0.2). Confounders considered at structural-level: university center, ID center. Clinical endpoint: mortality during the acute course of SARS-CoV-2 infection (deceased vs. not deceased)

**Fig. 4:**
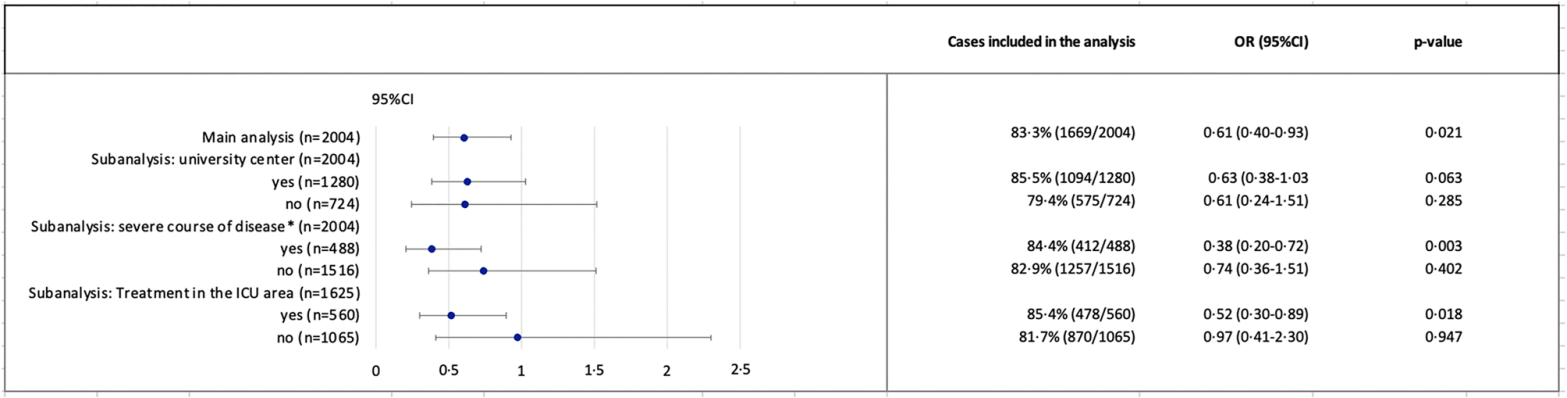
Effect of the structural characteristic ID center in the logistic regression analysis with the clinical endpoint mortality in different subgroups. Explanation: *CI95%* 95% confidence interval, *OR* odds ratio, *p-value* p-value for the confounder ID center in the multivariable analysis, *ID* infectious diseases, *ICU area* intensive care unit area. All other model assumptions and confounders in the subgroup analyses were considered according to the main analysis (Table 2). *Severe disease course: Need for catecholamines, life-threatening cardiac arrhythmias, unplanned mechanical ventilation (invasive or non-invasive), prolongation (> 24h) of planned mechanical ventilation, liver failure, qSOFA ≥2, renal failure on dialysis

In addition to these well-known patient-level risk factors, an independent effect of various structural characteristics was observed. The mortality risk of patients treated in a university center was higher than that of patients treated in non-university institutions (OR 1.63 (95% CI 1.05–2.54), p = 0.029). Patients treated in an ID center had a lower mortality risk compared to patients from non-ID centers (OR 0.61 (95% CI 0.40–0.93), p = 0.021). This association was also observed in various subgroup analyses (Fig. 4), among others for patients with a severe course of disease (n = 488, OR 0·38 (95% CI 0·20–0.72), p = 0.003). In further analyses, a treatment difference was observed in the group of patients with severe course of disease: 15·4% (69/449) of the patients received steroids, significantly more often in ID centers (20.5%, 62/302 vs. 4.8%, 7/147, p < 0.001).

## Discussion

Our study is the first to provide an overview of the current clinical ID care provision and the involvement of ID specialists in pandemic management and hospital care in Germany. It also demonstrates the advantages of ID care structures in the treatment of patients with COVID-19.

The study encompasses a wide range of hospitals of various sizes and levels of care. The characteristics of the study population appear to be representative for both German and international cohorts of hospitalized patients with COVID-19 [12–16]. The study population included cases from the wave of the COVID-19 pandemic in Germany and took into account established risk factors for severe disease progression.

Our results indicate that ID specialists have played a crucial role in pandemic management and inpatient care. In ID centers, ID specialists were always involved in the crisis management teams and in the inpatient care of COVID-19 patients. Furthermore, it is noteworthy to highlight the differences between ID centers and non-ID centers regarding their participation in treatment and therapy recommendations, clinical research as well as the organization and management of normal inpatient COVID-19 areas. These observations are also reflected internationally: in the USA, Australia, and New Zealand, it has been shown that ID specialists and microbiologists were crucially involved in the initial response to the pandemic. For example, they played a central role in coordinating local measures and ensuring the appropriate implementation of diagnostic tests [17–19]. At the same time, our results highlight the shortage of clinical ID specialists, which has been observed in recent decades: The estimated need for ID specialists according to Kern et al. was only achieved in about one third of the hospitals examined, including ID centers [20–22].

In terms of mortality, our study found clinical ID expertise to be a significantly protective factor in both univariate and multivariable models. This effect proved to be robust in subgroup analyses and was particularly strong in patients with severe course of disease. Furthermore, our data provide initial indications that an earlier and more consistent implementation of new treatment procedures (in this case: steroid administration) could be considered as potential factor influencing the mortality of COVID-19 patients. Over the past two decades, it has been shown that patients with severe infections in particular benefit from early involvement of ID specialists [23, 24]. In addition, improved quality of patient care, treatment outcomes, and use of antibiotics have been described [24, 25].

### Limitations

When assessing our study, limitations should be taken into consideration. The results at the structural level may only be partially representative of all German hospitals, even though a wide range of hospitals of various sizes and care levels were included. The active recruitment of patients within the framework of the LEOSS study was crucial for the selection of the hospital cohort examined. It is likely that this led to a bias in favour of the participation of hospitals with ID expertise and research interest. There are currently 29 DGI-certified centers in Germany (as March 14, 2022), with 14 of them participating in this study. Considering a total number of 1903 hospitals in Germany (Federal Statistical Office 2020), the nationwide proportion of ID centers is 1.5% (29/1903), compared to 35% (14/40) in our study [26, 27]. At the patient-level, the anonymous and nationwide recruitment allowed for the broad inclusion of patients, thereby reducing selection biases [28]. However, the anonymous and retrospective data collection resulted in limitations, as missing values or incomplete information could not be retrospectively obtained. In the conducted missing data analysis, no patterns were identified, and therefore, all potential confounders in our data set considered in the analyses. The literature describes additional risk factors such as a low socioeconomic status or genetic risk factors, which can influence mortality risk but were not captured with the LEOSS data set [29, 30].

In summary, our study provides important insights into the conditions under which patients were treated in German hospitals during the COVID-19 pandemic. These findings have important implications for health policy and care-related decisions. The ability of societies and healthcare systems to prepare for and respond effectively to a new ID depends on the planning, execution, and maintenance of emergency measures. Our study shows that clinical ID medicine has played a key role in this regard in the COVID-19 pandemic. An important and necessary step towards better preparedness for current and future pandemics is therefore the nationwide establishment of the new specialist training programme “Internal Medicine and Infectious Diseases” as well as the establishment of comprehensive clinical ID care provision. In our opinion, this is the only opportunity to improve the care situation for patients with ID in a structural and sustainable way in the long term.

## Supplementary Information

Below is the link to the electronic supplementary material.

Supplementary file1 (PDF 305 KB)
