# Supplementary material for: Role and benefits of infectious diseases specialists in the COVID-19 pandemic: Multilevel analysis of care provision in German hospitals using data from the Lean European Open Survey on SARS-CoV-2 infected patients (LEOSS) cohort
Source: Infection. 2024 Aug 16;53(1):259–69. doi: 10.1007/s15010-024-02362-2 (PMC11825572; doi:10.1007/s15010-024-02362-2)
Supplement: Supplementary file 1 — Supplementary file1 (PDF 305 KB) [file 15010_2024_2362_MOESM1_ESM.pdf]

Role and benefits of infectious diseases specialists in the COVID-19 pandemic - Multilevel analysis of care provision in German hospitals using data from the Lean European Open Survey on SARS-CoV-2 infected patients (LEOSS) cohort

Supplements

Supplement 1

Items			Mode of assessment		Item characteristics
Domain	Item	Scale level*	Entry Type	Scale	Structure indicator
**Section 1: Hospital characteristics, clinical infectious disease (ID) care provision (general section)**					
	Hospital name	String	Text field	Text	
External certification					
	Is your hospital a certified ID center of the German Society for Infectiology (DGI)?	Numeric	Tickbox (SC)	Yes/No	x
	Is your hospital a member or partner of the German Centre for Infection Research (DZIF)?	Numeric	Tickbox (MC)	Yes, member institution/Yes, associated partner/No	
	Is your hospital a competence and treatment centre of the Standing Working Group of Competence and Treatment Centres for high consequence ID (STAKOB)?	Numeric	Tickbox (SC)	Yes/No	
Staff/Team/Infrastructure					
	How many beds exist in your hospital in total?	String	Text field	Number	
	Are inpatient beds available in your hospital for patients receiving ID treatment?	Numeric	Tickbox (SC)	Yes, on a separate ward/Yes, as occupied beds/No	
	Does your hospital have explicit isolation rooms (room class II, negative pressure, airlock) according to DGKH guidelines for patients who release infectious aerosols?	Numeric	Tickbox (SC)	Yes/No	
	How many specialist departments are there in your hospital?	String	Text field	Number	
	Does your hospital have an ID department?	Numeric	Tickbox (SC)	Yes, as an independent clinic/Yes, as part of another clinic/No	
	Is there a staff unit/central unit for ID?	Numeric	Tickbox (SC)	Yes, as an independent institute/Yes, together with hospital hygiene/Yes, together with another speciality (not hospital hygiene)/No	
	Does the hospital have a department or staff unit for ID in which at least two ID physicians work who are qualified as ID specialists according to the German Society for Infectiology (DGI) or state medical association?	Numeric	Tickbox (SC)	Yes/No	x
	Is there an ABS team according to the S3 guideline "Strategies to ensure rational antibiotic use in hospitals"?	Numeric	Tickbox (SC)	Yes/No	x
	Is there an ABS team in an alternative organisational form?	Numeric	Tickbox (SC)	Yes/No	
	There is no department, staff unit, ABS team or ID consultation service.	Numeric	Tickbox (SC)	Yes/No	
	If there is no department, staff unit or ID consultant service, how is ID expertise organised alternatively?	Numeric	Tickbox (MC)	External infectious disease service providers (e.g. laboratories or infectious disease specialists in private practice)/microbiological consults/virological consults/consults by hospital hygiene/personal network of attending physicians/other organisation	
	If there is a department or staff unit for ID, how many ID specialists (according to the State Medical Association or DGI) work there?	String	Text field	Number	
	Has the estimated need for ID specialists per bed number according to Kern et al. been reached?	Numeric	Tickbox (SC)	Yes/No	x
	Does your hospital have a diagnostic laboratory with availability of all modern detection methods for the diagnosis of infectious agents and infections (DIN-certified)?	Numeric	Tickbox (SC)	Yes/No	
	How many inpatients are seen by the ID department in your hospital per year?	String	Text field	Number	
	How many outpatients are seen by the ID department in your hospital per year?	String	Text field	Number	
Further education and training					
	Is your hospital authorised to provide further training in ID according to the State Medical Association?	Numeric	Tickbox (SC)	Yes/No	
	Are ID training courses (by ID experts) held regularly for physicians and medical assistants?	Numeric	Tickbox (SC)	Yes, weekly/Yes, monthly/No	
Outpatient availability					
	Are there ID outpatient areas in your hospital?	Numeric	Tickbox (MC)	Yes, an interdisciplinary infection outpatient clinic/Yes, an HIV outpatient clinic/Yes, a TB outpatient clinic/Yes, a travel medicine outpatient clinic/Yes, an outpatient clinic for tropical diseases/Yes, an outpatient clinic for parasitoses or echinococcoses/Yes, other/Yes, a COVID-19 outpatient clinic/No	
	If there are ID outpatient areas in your hospital, by which department are they managed?	Numeric	Tickbox (SC)	Outpatient clinics are under the direction of the Clinic for ID/outpatient clinics are under the direction of another clinic	
Consultative availability					
	Is there an ID consultation service?	Numeric	Tickbox (SC)	Yes/No	x
	If there is an infectious disease consultation service, how many infectious disease consults are performed per year?	String	Text field	Number	
	If there is an ID consultant service, how many stop-order consultations are carried out per year?	String	Text field	Number	
	If there is an ID consultation service, where do the consultations take place?	Numeric	Tickbox (MC)	At the bedside/Telephone/Written	
	If there is an ID consultation service, do ID consultations take place mainly at the bedside?	Numeric	Tickbox (SC)	Yes/No	x
Information Technology					
	If there is an infectious disease consultation service, how is it requested?	Numeric	Tickbox (MC)	Request for infectious disease consultation service manually by telephone, fax, e-mail or other internal message structures/Request for infectious disease consultation service automatically in case of pathogen detection in blood culture/Request for infectious disease consultation service automatically in case of prescription of reserve anti-infectives/Request for infectious disease consultation service automatically in case of positive SARS-CoV-2 smear/Request for infectious disease consultation service automatically in case of a COVID-typical radiological finding.	
**Section 2: Structure and organisation of care in the pandemic in March/April 2020 (COVID-19-specific)**					
	How many COVID-19 patients were treated as inpatients in your hospital in the period 01.03. - 30.04.2020?	String	Text field	Number	
	How many COVID-19 patients were treated as outpatients in your hospital in the period 01.03. - 30.04.2020?	String	Text field	Number	
	What was the maximum number of beds available in your hospital for COVID-19 patients?	String	Text field	Number	
	How many of the explicit isolation rooms (room class II, negative pressure, airlock) according to the DGKH guideline were used for COVID-19 patients?	String	Text field	Number	
	Which departments and divisions were involved in the crisis team?	Numeric	Tickbox (MC)	Anaesthesiology/Surgery/Gastroenterology/Haematology/Oncology /Hygiene and Environmental Medicine/Internal Medicine/Infectiology/Infection Epidemiology/Internal Medicine/Paediatrics and Adolescent Medicine/Microbiology/Nephrology/Nurology/Palliative Medicine/Pneumology/Cardiology/Transfusion Medicine/Virology/Nursing Services and Adolescent Medicine/Microbiology/Nephrology/Neurology/Palliative Medicine/Pneumology/Cardiology/Transfusion Medicine/Virology/Nursing Service Management/Department of Personnel/Department of Organisation and Infrastructure/Department of Procurement/Department of Quality Management/Department of Finances/Department of Costs/Other/There was no Crisis Team	
	Were there any designated testing centres (e.g. tents or containers) for SARS-CoV-2 testing on or near the hospital site?	Numeric	Tickbox (SC)	Yes/No	
	Was there a separate COVID-19 outpatient area on the hospital campus?	Numeric	Tickbox (SC)	Yes/No	
	Was there strict separation of patient care in three inpatient areas according to RKI recommendations (SARS-CoV-2 positive patients, suspected cases and SARS-CoV-2 negative patients)?	Numeric	Tickbox (SC)	Yes/No	
	Was there a separate suspected case area for COVID-19 patients?	Numeric	Tickbox (SC)	Yes, separate ward/Yes, separate area on a ward/No	
	Was there a separate normal inpatient area for COVID-19 patients?	Numeric	Tickbox (SC)	Yes, separate ward/Yes, separate area on a ward/No	
	Was there a separate IMC area for COVID-19 patients?	Numeric	Tickbox (SC)	Ja, eigene Station/Ja, eigenen Bereich auf einer Station/Nein	
	Was there a separate ICU area for COVID-19 patients?	Numeric	Tickbox (SC)	Yes, separate ward/Yes, separate area on a ward/No	
	Was there a separate area for paediatric COVID-19 patients?	Numeric	Tickbox (SC)	Yes, separate ward/Yes, separate area on a ward/No	
	Was there a separate area for palliative COVID-19 patients?	Numeric	Tickbox (SC)	Yes, separate ward/Yes, separate area on a ward/No	
	Was there separate staff for SARS-CoV-2 positive patients, suspected cases and SARS-CoV-2 negative patients?	Numeric	Tickbox (SC)	Yes, there was a strict allocation of medical staff to three patient groups/Yes, there was a strict allocation of medical staff to two patient groups/Yes, other/No	
	Was there a delay in important examinations (interventions and functional diagnostics)?	Numeric	Tickbox (SC)	Yes, in all patients/Yes, but only in SARS-CoV-2 positive patients/No	
	To what extent could care services (consults) for COVID-19 patients take place?	Numeric	Tickbox (SC)	Offer regularly continued/Offer continued with restrictions/Offer completely discontinued/Service not offered	
	To what extent could care services (physiotherapy) take place for COVID-19 patients?	Numeric	Tickbox (SC)	Offer regularly continued/Offer continued with restrictions/Offer completely discontinued/Service not offered	
	To what extent could care services (speech therapy) take place for COVID-19 patients?	Numeric	Tickbox (SC)	Offer regularly continued/Offer continued with restrictions/Offer completely discontinued/Service not offered	
	To what extent could care services (psychological support) take place for COVID-19 patients?	Numeric	Tickbox (SC)	Offer regularly continued/Offer continued with restrictions/Offer completely discontinued/Service not offered	
	To what extent could care services (palliative care) take place for COVID-19 patients?	Numeric	Tickbox (SC)	Offer regularly continued/Offer continued with restrictions/Offer completely discontinued/Service not offered	
	To what extent could care services (optional surgeries) take place for COVID-19 patients?	Numeric	Tickbox (SC)	AOffer regularly continued/Offer continued with restrictions/Offer completely discontinued/Service not offered	
	Has a notifiable nosocomial outbreak occurred with regard to SARS-CoV-2?	Numeric	Tickbox (SC)	Yes/No	
	In the case of severely ill (SARS-CoV-2) patients, was contact made with another centre for individual case discussion and advice on possible further therapies?	Numeric	Tickbox (SC)	Yes/No	
	Were severely ill (SARS-CoV-2) patients transferred to another centre?	Numeric	Tickbox (SC)	Yes/No	
	Has your hospital participated or is participating in interventional trials of new treatments for COVID-19 patients?	Numeric	Tickbox (SC)	Yes/No	
	Has your hospital participated or is participating in COVID-19 trial registries other than LEOSS?	Numeric	Tickbox (SC)	Yes, on one/Yes, on two/Yes, on more than two/No	
	Has biomaterial been collected from COVID-19 patients in your hospital?	Numeric	Tickbox (SC)	Yes/No	
**Section 3: Involvement of clinical ID specialists in pandemic management and care in March/April 2020 (COVID-19-specific part)**					
	How many beds were available in the normal inpatient area for COVID-19 patients?	String	Text field	Number	
	Were ID specialists involved in patient care and therapy in the normal inpatient setting?	Numeric	Tickbox (SC)	Yes/No	
	How were ID specialists involved in the normal inpatient area for COVID-19 patients?	Numeric	Tickbox (SC)	Head of department/regular ID rounds/ID consults as required/other involvement/no involvement	
	Was the normal inpatient area led by ID specialists?	Numeric	Tickbox (SC)	Yes/No	x
	How many beds were available in the IMC area for COVID-19 patients?	String	Text field	Number	
	Were ID specialists involved in patient care and therapy in the IMC area?	Numeric	Tickbox (SC)	Yes/No	
	How was the involvement of ID specialists in the IMC area for COVID-19 patients?	Numeric	Tickbox (SC)	Head of department/regular ID rounds/ID consults as required/other involvement/no involvement	
	Were there regular infectious disease visits in the IMC area?	Numeric	Tickbox (SC)	Yes/No	x
	How many beds were available in the ICU area for COVID-19 patients?	String	Text field	Number	
	Were ID specialists involved in patient care and therapy in the ICU area?	Numeric	Tickbox (SC)	Yes/No	
	How were ID specialists involved in the ICU area for COVID-19 patients?	Numeric	Tickbox (SC)	Head of department/regular ID rounds/ID consults as required/other involvement/no involvement	
	Were there regular infectious disease visits in the ICU area?	Numeric	Tickbox (SC)	Yes/No	x
	Were ID specialists involved in the organisation of outpatient and prehospital areas within the framework of the crisis team?	Numeric	Tickbox (SC)	Yes/No	
	Were ID specialists involved in the organisation of inpatient COVID-19 areas within the framework of the crisis team?	Numeric	Tickbox (SC)	Yes/No	
	Were ID specialists involved in recommending treatment and therapy within the framework of the crisis team?	Numeric	Tickbox (SC)	Yes/No	x
	Were infectiologists involved in the planning of studies within the framework of the crisis team?	Numeric	Tickbox (SC)	Yes/No	
	Which departments were involved in the care of patients in the normal inpatient area?	Numeric	Tickbox (MC)	Anaesthesiology/Surgery/Gastroenterology/Haemat-Oncology/Hygiene and Environmental Medicine/Internal Medicine/Infectiology/Infection Epidemiology/Internal Medicine/Paediatrics/Microbiology/Nephrology/Neurology/Palliative Medicine/Pneumology/Cardiology/Transfusion Medicine/Virology/Others	
	Other?	String	Text field		
	Which departments were involved in the care of patients in the IMC area?	Numeric	Tickbox (MC)	Anaesthesiology/Surgery/Gastroenterology/Haemat-Oncology/Hygiene and Environmental Medicine/Internal Medicine/Infectiology/Infection Epidemiology/Internal Medicine/Paediatrics/Microbiology/Nephrology/Neurology/Palliative Medicine/Pneumology/Cardiology/Transfusion Medicine/Virology/Others	
	Other?	String	Text field		
	Which departments were involved in the care of patients in the ICU area?	Numeric	Tickbox (MC)	Anaesthesiology/Surgery/Gastroenterology/Haemat-Oncology/Hygiene and Environmental Medicine/Internal Medicine/Infectiology/Infection Epidemiology/Internal Medicine/Paediatrics/Microbiology/Nephrology/Neurology/Palliative Medicine/Pneumology/Cardiology/Transfusion Medicine/Virology/Others	
	Other?	String	Text field		

***Legend: (a) String: score as free text field(metric values or text), will be categorised and/or censored before export; (b) Numeric: categorical (qualitative) score either as single response (SC) or multiple response (MC).**

Supplement 2

Methods - Statistical analyses at patient-level

In the multivariable model, the potential confounders age, gender, comorbidities, stage of disease at diagnosis and the structural characteristics University and Infectious diseases (ID) center were included on the basis of a literature search (Medline: PubMed, LitCovid) and the availability in the database (Table S1).

Table S1: Categorisation of the dependent variable and independent variables (potential confounders) included in the univariate and multivariable logistic regression analysis.

**Items**	**Form of assessment**	
	Scale level	Scale
**Dependent variable**		
Mortality	Nominal (dichotomous)	1=deceased, 0=not deceased
**Independent variable**		
**Age (years)**	Nominal	1= <=45, 2= 46-65, 3= 66-85, 4= >85
**Gender**	Nominal	1=Male, 2=Female
**Comorbidities**[1]		
Cardiovascular diseases	Nominal (dichotomous)	1=Yes, 0=No
Diabetes mellitus	Nominal (dichotomous)	1=Yes, 0=No
Chronic pulmonary disease	Nominal (dichotomous)	1=Yes, 0=No
Haematological or oncological disease	Nominal (dichotomous)	1=Yes, 0=No
Renal disease	Nominal (dichotomous)	1=Yes, 0=No
Neurological disease	Nominal (dichotomous)	1=Yes, 0=No
**Stage of disease at diagnosis**[2]	Nominal	1=Uncomplicated, 2=Complicated, 3=Critical
**Body-Mass-Index (kg/m2)**	Nominal	1= <18·5, 2= 18·5-24·9, 3= 25-29·9, 4= 30-34·9, 5= >34·9
**Characteristics of the hospitals**		
Universitary center	Nominal (dichotomous)	1=Yes, 0=No
ID center	Nominal (dichotomous)	1=Yes, 0=No

Explanation: [1]no reference group; Comorbidities were dichotomised as cardiovascular disease (myocardial infarction, aortic stenosis, AV block, carotid artery disease, chronic heart and circulatory failure, peripheral vascular disease, hypertension, atrial fibrillation, coronary heart disease), chronic pulmonary disease (chronic obstructive pulmonary disease, asthma, other chronic lung diseases), haematological or oncological disease (leukaemia, lymphoma, solid tumours, stem cell transplantation), diabetes mellitus (with and without end-organ damage), renal disease (acute kidney injury at the time of SARS-CoV-2 detection, chronic kidney disease), neurological disease (hemiplegia, dementia, cerebrovascular disease, stroke, transient ischaemic attack, motor neurone disease, movement disorders, multiple sclerosis, myasthenia gravis, neuromyelitis optica spectrum disorder, other neurological autoimmune diseases, other pre-existing neurological conditions). A comorbidity was defined as present if at least one specific comorbidity was documented.
[2]Uncomplicated: Asymptomatic, symptoms of upper respiratory tract infection, nausea, vomiting, diarrhoea, fever; Complicated: new oxygen requirement, relevant increase in prior oxygen therapy, paO_2 on room air <70mmHg, SO_2 on room air <90%, GOT or GPT >5x ULN, new cardiac arrhythmia, new pericardial effusion >1cm, new heart failure with pulmonary oedema, congestive hepatopathy or peripheral oedema; Critical: Need for catecholamines, life-threatening arrhythmias, unplanned mechanical ventilation (invasive or non-invasive), prolongation (>24h) of planned mechanical ventilation, liver failure, qSOFA >=2, renal failure requiring dialysis.

The included confounders considered for the analyses at patient-level are baseline characteristics. Time-dependent covariates were not included in the analysis. In preparation for the regression model, multicollinearity of the confounders taken into account could be excluded with a variance inflation factor <10. The Nagelkerkes R-squared was considered to examine possible and identify the best fitting model. In the final multivariable regression model, all variables were included with a univariate significance level ≤0·2. We calculated the odds ratios (OR) with the 95% confidence interval (CI).
In the course, different subgroup analyses were performed: Firstly, the influence of confounders with more than 5% missing values (body mass index, neurological disease) was examined. The missing analyses showed that the results were robust with and without these confounders. All missing values could be classified as missing-completely-at-random.[1] The results of the subgroup analysis for the confounder body mass index are shown in Table S2.

Table S2: Subgroup analysis of cases in which the confounder body mass index (kg/m2) was recorded (n=1085): Multivariable logistic regression analyses with the clinical endpoint mortality with and without the confounder body mass index.

		**Multivariable analysis without Body-Mass-Index**		**Multivariable analysis with Body-Mass-Index**	
	All patients	OR (95%CI)	p-value	OR (95%CI)	p-value
**Total**	1085				
**Age**					
<=45		0·03 (0·01-0·10)	**<0·001**	0·03 (0·01-0·10)	**<0·001**
46-65		0·09 (0·05-0·18)	**<0·001**	0·09 (0·05-0·18)	**<0·001**
66-85		0·35 (0·20-0·61)	**<0·001**	0·34 (0·20-0·60)	**<0·001**
>85		Ref.		Ref.	
**Gender**					
Female		Ref.		Ref.	
Male		1·38 (0·95-2·01)	0·090	1·37 (0·94-2·00)	0·107
**Comorbidities**					
Cardiovascular diseases		1·09 (0·69-1·72)	0·703	1·06 (0·67-1·69)	0·802
Diabetes mellitus		0·90 (0,59-1·38)	0·633	0·90 (0·59-1·38)	0·617
Chronic pulmonary disease		1·34 (0·84-2·14)	0·215	1·35 (0·85-2·16)	0·206
Haematological or oncological disease		1·26 (0·81-1·96)	0·313	1·27 (0·81-1·99)	0·293
Renal disease		1·84 (1·22-2·76)	**0·003**	1·85 (1·23-2·79)	**0·003**
**Stage of disease at diagnosis**					
Uncomplicated		Ref.		Ref.	
Complicated		2·98 (2·02-4·40)	**<0·001**	2·97 (2·01-4·38)	**<0·001**
Critical		12·35 (7·06-21·59)	**<0·001**	12·20 (6·93-21·48)	**<0·001**
**Body-Mass-Index**					
<18·5		*	*	0·80 (0·23-2·80)	0·730
18·5-24·9		*	*	Ref.	
25-29·9		*	*	1·13 (0·74-1·73)	0·577
30-34·9		*	*	1·00 (0·58-1·72)	0·994
>34·9		*	*	1·13 (0·58-2·20)	0·724
**Characteristics of the hospitals**					
University center		1·82 (0·91-3·61)	0·089	1·82 (0·91-3·62)	0·090
ID center		0·51 (0·26-0·99)	**0·048**	0·51 (0·26-1·00)	0·051

Explanation: *CI*95% 95% confidence interval, *OR* odds ratio, *ID* Infectious diseases, *Ref* reference category, *p-value* p-value for multivariable analysis. Confounders considered at patient-level: age (in years), gender, comorbidities (no reference group, comorbidities were dichotomised, see Table S1), stage of disease at diagnosis (see Table S1), body mass index (in kg/m2, *analysis without the confounder BMI). Confounders considered at structural-level: university centre, ID centre. Clinical endpoint: mortality during the acute course of SARS-CoV-2 infection (deceased vs. not deceased).

In addition, we performed subgroup analyses to check the robustness of our results. Therefore, the influence of the structure characteristic ID center was analyzed in the logistic regression analysis with the clinical endpoint mortality in different subgroups (Figure A4).

The structure characteristics included as variables in the multivariable model were collected at the hospital level. Patient care and hospital structures have been subject to constant change since the beginning of the pandemic and were adapted to the changing situations depending on the local situation and the course of the pandemic. In order to prevent the results from being distorted by this temporal component, a period was specifically selected for the data collection at both the structure- and patient-level, from which it could be assumed that the conditions in the hospitals were as stable as possible. Based on the number of COVID-19 cases in Germany reported to the Robert Koch Institute (RKI), the time period from 1 March 2020 to 30 April 2020 was selected, in which the maximum of the first wave of infections occurred.[2]

All statistical analyses were performed using IBM® SPSS® statistical software, version 28·0 (Released 2021, Armonk, NY: IBM Corp).

References

1. Carpenter JR, Smuk M. Missing data: A statistical framework for practice. *Biometrical Journal* 2021; **63**(5): 915-47.
2. Robert Koch Institut (RKI). COVID-19 (Coronavirus SARS-CoV-2). https://www.rki.de/DE/Content/InfAZ/N/Neuartiges_Coronavirus/nCoV.html (accessed 08.11.2021 2020).
